# 

**Published:** 1896-08

**Authors:** 


### CONTENTS—AUGUST, 1896.—VOL. XII., NO. 2.

ORIGINAL COMMUNICATIONS—

Broncho-Pneumonio. N. A. Olive, M. D., Waco, Texas . . . . . . 57
Treatment of Abortion. S. W. Long, M. D., Richmond, Va . . . . 62
Value of Abdominal Belt After Cœliotomies. Stuart McGuire, M. D., Richmond, Va . . . . . . . . . . . . . . . . . . . . . . . . 64
A New Stone Searcher. Lucien Lofton, M. D., Atlanta, Ga . . . . 69
Umbilical Hemorrhage. H. B. Granberry, M. D., Austin . . . . 70

ABSTRACTS AND SELECTIONS—

Relation of Examining Boards to State, etc . . . . . . . . . . 76
New Cure for Gonorrhea . . . . . . . . . . . . . . . . . . . . 77

SOCIETY NOTES—

Pan-American Medical Congress . . . . . . . . . . . . . . . . 78
Vivisectionists Scared Up . . . . . . . . . . . . . . . . . . 81
East Texas Medical Society. Quarterly Meeting . . . . . . . . . 82

EDITORIAL—

Abortion of Small Pox . . . . . . . . . . . . . . . . . . . . 83
The Worm Turns . . . . . . . . . . . . . . . . . . . . . . . 84
"Told You So" . . . . . . . . . . . . . . . . . . . . . . . . 87
Gems of Journal Literature . . . . . . . . . . . . . . . . . . 88
Some Further Perversion of Facts . . . . . . . . . . . . . . . 90
The Texas Medical News' "Explanation," etc . . . . . . . . . . 91
Quackery Rampant . . . . . . . . . . . . . . . . . . . . . . 94

MEDICAL NEWS AND MISCELLANY—

Numerous Items . . . . . . . . . . . . . . . . . . . . . . . 99

THE JOURNAL'S OWN PAGE . . . . . . . . . . . . . . . . . . . . 102
PUBLISHERS' NOTES . . . . . . . . . . . . . . . . . . . . . . 103

